# Impact of physical distancing policy on reducing transmission of SARS-CoV-2 globally: Perspective from government’s response and residents’ compliance

**DOI:** 10.1371/journal.pone.0255873

**Published:** 2021-08-10

**Authors:** Ping-Chen Chung, Ta-Chien Chan

**Affiliations:** 1 Department of Dentistry, Puzi Hospital, Ministry of Health and Welfare, Chiayi, Taiwan; 2 Research Center for Humanities and Social Sciences, Academia Sinica, Taipei, Taiwan; 3 Institute of Public Health, School of Medicine, National Yang Ming Chiao Tung University, Taipei, Taiwan; King Faisal University College of Veterinary Medicine and Animal Resources, SAUDI ARABIA

## Abstract

**Background:**

COVID-19 was declared a public health emergency by the World Health Organization (WHO) in January 2020. Various physical distancing interventions were introduced to flatten the epidemic curve and reduce the disease burden. We evaluated the impacts of policy stringency and residents’ compliance on time-varying reproduction number in 17 countries.

**Methods:**

Data were from WHO reports of local transmission (February 28 to April 8, 2020) in Australia, Canada, Finland, France, Germany, Greece, Italy, Spain, Sweden, Thailand, the UK, US and Vietnam. Earlier local transmission data where available from press releases were added for Japan, South Korea, Singapore and Taiwan starting January 28, 2020. COVID-19 policy responses were from the Oxford Covid-19 Government Response Tracker with 17 indicators. Changes in people’s behaviors were from Google’s COVID-19 community mobility reports and Apple Maps’ mobility trends reports. We estimated the daily time-varying reproduction number (Rt) by country. 0-, 7- and 14-day lagged effects of non-pharmaceutical interventions and changes in human mobility on Rt were estimated by linear mixed-effects models.

**Results:**

Rt initially surged rapidly, then declined gradually depending on policy stringency. The highest mean policy stringency scores were for Italy (69.97) and South Korea (61.00). Variations in stringency scores were higher in Europe, the US and Australia than in Asia. The human mobility reduction was greater in countries with strict policies (median stringency score > = 50). In terms of immediate (0-day lag) effects, Rt reductions were found for workplace-closure, limited-gathering, and stay-at-home policies. At a 7-day lag, Rt reductions were found for workplace closure, restrictions on gatherings, stay-at-home requirements, international travel controls, contact tracing and reducing walking around. At a 14-day lag, Rt reductions were found for restrictions on gatherings, less visiting and staying in parks, and reduced walking around.

**Conclusion:**

The findings show physical distancing policies and residents’ compliance can slow transmission, with the lag-to-effect time varying by policy.

## Introduction

Emerging, highly contagious SARS-CoV-2 has spread out globally since the large outbreak in January 2020 in China [1] and has caused a global COVID-19 pandemic, for which there are no effective, safe pharmaceutical agents for treatment or prevention. Non-pharmaceutical intervention (NPI) is the only public health approach to reduce transmission, control the pandemic and lower the disease burden. However, in the past few decades, people have not experienced extensive city lockdowns or physical distancing (also known as social distancing) policies. In some epidemics of influenza or enterovirus, some public health authorities have implemented school closures [2] or temporary class suspensions [3], but rarely for a whole city. Unprecedented physical distancing policies have been implemented to cope with the COVID-19 pandemic in different countries [4]. Due to the huge socio-economic impacts from control policies, governments in different countries have set different stringency levels for physical distancing policies, taking into consideration feasibility [5], the culture [6], medical capacity [7], social acceptance [6], and economic loss [6], and such policies have also evolved with the pandemic situation.

In order to control the pandemic, physical distancing policies have been introduced to reduce the chance of interpersonal contact and transmission. Unlike seasonal influenza, the asymptomatic infection rate has been estimated to be as high as 20.8% for SARS-CoV-2 [8], and the virus also has higher transmissibility as measured by the reproduction number (R0, ranging from 2 to 3) [1, 9] than seasonal influenza (R0: 1.19–1.37) [10]. In addition, epidemiological investigation has found that transmission occurs before the onset of symptoms [11]. These factors, as well as similarity of the symptoms to those of influenza, all increase the barrier to early detection of the disease. The different physical distancing policies have lowered the effective reproduction number (Rt) over time, aiming to interrupt the chain of transmission and maintain the Rt below 1.

The effectiveness of physical distancing policies depends on their stringency and public compliance. A previous simulation study showed that social distancing intervention starting earlier in an epidemic could delay the epidemic curve, and intervention introduced later could flatten the epidemic curve [7]. Empirically, the effectiveness of NPIs including travel restrictions, stay-at-home policies, school closures, cancelling public gatherings and so on has been reported on in different countries. In contrast to NPIs, public compliance affects the probability of transmission. With the technology of mobile devices, changes in human mobility can be measured in an aggregated fashion. Those data reflect human movements during an epidemic period under different physical distancing policies.

This paper will primarily focus on only confirmed cases based on local transmission, which is a fair way to measure the policy effectiveness. The effective reproduction number (Rt) was used to measure transmissibility, based on time after introducing particular physical distancing interventions. In the real world, government policies and residents’ compliance occur at the same time. In this study, we visualize the Rt and policy stringency in a spatio-temporal manner. We then compare the strategies of different NPIs and changes in human mobility in different countries. Finally, we model the effectiveness of NPIs and residents’ compliance on reducing Rt with different lagged effects.

## Methods

### Data source

A confirmed case of COVID-19 infection was defined as a person with laboratory confirmation, irrespective of clinical signs and symptoms. All the data were captured from novel coronavirus (2019-nCoV) situation reports published by the World Health Organization (https://www.who.int/emergencies/diseases/novel-coronavirus-2019/situation-reports). The data were updated daily starting January 21, 2020 and covered the situation of COVID-19 infection worldwide. The WHO Novel Coronavirus (2019-nCoV) SITUATION REPORT presented transmission classification starting from February 28, 2020. Before that date, the WHO reported confirmed cases containing local and imported cases. From February 28, 2020 to April 8, 2020, daily reported laboratory-confirmed cases of COVID-19 included transmission classification based on WHO analysis of available official data, and may be subject to reclassification as additional data become available. We extracted the data classified as “local transmission” in this study. Therefore, we searched for data from local sources on daily confirmed cases on each country’s official website, found South Korea, Japan, and Singapore reported daily new local confirmed cases, and added these data into the analysis. In addition, Taiwan’s daily local cases were not reported from the WHO, so we collected data between January 28, 2020 and April 8, 2020 from official press releases of the Taiwan CDC. We obtained Taiwan’s daily confirmed, locally transmitted cases of COVID-19 from January 28, 2020 to April 8, 2020 from announcements by the Taiwan Centers for Disease Control (https://www.cdc.gov.tw/En/Bulletin/List/7tUXjTBf6paRvrhEl-mrPg). We took South Korea daily confirmed, locally transmitted cases from the Korea Centers for Disease Control and Prevention (http://ncov.mohw.go.kr/tcmBoardList.do?brdId=3) from January 30, 2020 to February 27, 2020. We counted the daily confirmed, locally transmitted cases of COVID-19 from press releases by the Ministry of Health Labor and Welfare (https://www.mhlw.go.jp/stf/houdou/index.html) during January 28, 2020 to February 27, 2020 in Japan and from the Ministry of Health (https://www.moh.gov.sg/news-highlights) during February 4, 2020 to February 27, 2020 in Singapore. The data included 17 different countries (regions)—Australia, Canada, Finland, France, Germany, Greece, Italy, Japan, South Korea, Singapore, Spain, Sweden, Taiwan, Thailand, the United Kingdom, United States and Vietnam—around the world, with few missing values during the study period.

### Oxford COVID-19 Government Response Tracker [12]

The Oxford COVID-19 Tracker collects publicly available information on 17 indicators such as containment and closure, as well as economic and health system policies from more than 160 countries to track and compare policy responses around the world. The stringency index was calculated using eight policy indicators (C1-C8) recording information on containment and closure policies, and one indicator (H1) recording health system policies (each indicator’s coding is provided on https://github.com/OxCGRT/covid-policy-tracker/blob/master/documentation/codebook.md. The indicators include C1 School closing, C2 Workplace closing, C3 Cancel public events, C4 Restrictions on gatherings, C5 Close public transport, C6 Stay at home requirements, C7 Restrictions on internal movement, C8 International travel controls and H1 Public information campaigns.). The daily value of the index is the average of nine sub-indices ranging between 0 and 100. We obtained the dataset from January 1, 2020 to April 8, 2020.

### COVID-19 Community Mobility Reports [13]

This dataset shows daily percentage changes in people’s visits to and staying time in six categories of places compared to a baseline which is the median value for the corresponding day of the week during the five-week period from January 3 to February 6, 2020. The categories for grouping places with similar characteristics for purposes of social distancing guidance include grocery stores and pharmacies, parks, transit stations, retail and recreation, residences, and workplaces. We used the data from February 15, 2020 to April 8, 2020.

### Mobility Trends Reports using information collected from Apple Maps [14]

The daily Mobility Trends Reports reflect changes in requests for directions by transportation type including driving, using public transport and walking for all available countries, regions and cities compared to a baseline volume on January 13, 2020 in Apple Maps in order to learn about COVID-19 mobility trends. We used the data from January 13, 2020 to April 8, 2020 after normalizing the data by day of the week effects. We further used these changes to normalize the different days of week. We took all mobility changes on Monday to conduct normalization and continued this process from Tuesday to Sunday. The purpose of this normalization is to consider the periodic pattern of human mobility on specific days of the week.

### Estimation of time-varying reproduction number (Rt)

The serial interval of COVID-19 is the time between a primary case’s (infector’s) clinical onset and secondary case’s (infectee’s) clinical onset. Du et al. estimated the distribution of the serial interval from 468 confirmed cases of COVID-19 reported in 93 Chinese cities by February 8, 2020 [15]. Following the estimated results from Du et al., we assumed a gamma distribution for the serial interval with a mean of 3.96 and a standard deviation of 4.75 days, and calculated time-dependent daily reproduction numbers over a seven-day moving window.

Cori et al. suggested fulfilling the following three criteria on estimating Rt: at least after the time window, at least after one mean serial interval, and when at least 12 cases have been observed since the beginning of the epidemic [16]. Thus, we didn’t take the beginning reproduction number into account until we met the above criteria in Taiwan, South Korea, Japan and Singapore. The data of other countries were left truncated starting from February 28, 2020, and their reproduction numbers met the criteria of at least after the time window and at least after one mean serial interval. The time-dependent daily reproduction numbers were computed using R 4.0.0 (R Core Team) and the EpiEstim (v2.2–1) package.

### Policy timing

The policy time was regarded as the time interval between the first confirmed COVID-19 case, whether local or imported, and the beginning of the government’s response according to the stringency index.

### Lagged effect on non-pharmaceutical interventions and personal protective behavior

We assumed that NPIs and individual behavioral change could influence the time-varying reproduction number without delay, and with delays of 7 days and 14 days. The reasons for the chosen delay periods were as follows. First, the incubation period of COVID-19 has been estimated at around 5.2 days [17]. Second, a test for COVID-19 is usually performed after the onset of symptoms, and it takes time to get the test results. Third, people need time to adjust to the intervention and change their behavior. Thus, we did 0-day, 7-day and 14-day lagged effects as sensitivity analysis.

### Statistical analysis

The Mobility Trends Reports on using public transportation collected from Apple Maps had more than 20% missing values (n = 150), and this variable was excluded in the following analysis. The variables of income support, debt or contract relief, fiscal measures, international support, emergency investment in healthcare, and investment in vaccines were excluded due to a large percentage of missing values at the early stage of the pandemic. We stratified different countries into two groups based on the median stringency index of 50. After examining the normality of the distribution, we used a random effects model with a random intercept to compare the difference in people’s behavioral changes across two different stringency groups, and to figure out the relationship between policy stringency and people’s behavioral changes. The variables of the stringency index and community mobility trends were evaluated in multivariable models using stepwise selection to eliminate those with p values >0.1 after excluding multicollinearity (variance inflation factor (VIF) > 10). The missing data including debt or contract relief (E2, n = 14), fiscal measures (E3, n = 6), emergency investment in healthcare (H4, n = 30), investment in vaccines (H5, n = 4) and community mobility data (n = 14) were excluded. We used linear mixed-effects models to explore the daily reproduction number with countries as a random effect. We also estimated 7- and 14-day lagged effects of policy on the time-varying reproduction number. All statistical tests were two-sided with a significance level of 0.05. All analyses were performed with R 4.0.0 statistical software. We used ArcGIS (ArcMap, version10.3; ESRI Inc., Redlands, CA, USA) and our developed ringmap toolbox (https://www.esri.com/about/newsroom/arcuser/looking-at-temporal-changes) to visualize the daily local confirmed cases, Rt and stringency scores in 17 countries during the study period. The world base map is downloaded from a publicly available website (http://thematicmapping.org/downloads/, CC BY-SA 3.0).

## Results

The earliest implementation of NPI was 21 days before the first case was confirmed. International travel controls (C8), public information campaigns (H1) and testing policy (H2) were implemented early in Singapore. And the latest implementation of NPI was 53 days after the first case was confirmed (Table 1). The median of the stringency index, which represents strictness of policies, was 52.78 (*interquartile range* = 43.12), the loosest stringency index was 2.78, and the strictest stringency index was 97.35 during the study period.

**Table 1:** Descriptive statistic of the explanatory variables.

Variables	N	Min	Q1	Q2	Q3	Max
Policy timing	635	-21	-9	0	8	53
**Oxford Covid-19 Government Response Tracker**						
Stringency Index	635	2.78	32.01	52.78	75.13	97.35
**Containment and closure policies**						
C1_School closing	635	0	0	3	3	3
C2_Workplace closing	635	0	0	1	2	3
C3_Cancel public events	635	0	1	2	2	2
C4_Restrictions on gatherings	635	0	0	2	4	4
C5_Close public transport	635	0	0	0	1	2
C6_Stay at home requirements	635	0	0	1	2	3
C7_Restrictions on internal movement	635	0	0	1	2	2
C8_International travel controls	635	0	2	3	4	4
**Economic policies**						
E1_Income support	635	0	0	0	2	2
E2_Debt or contract relief	621	0	0	0	1	2
E3_Fiscal measures (1000000USD)	629	0	0	0	0	1960000
E4_International support (1000000USD)	567	0	0	0	0	834000
**Health system policies**						
H1_Public information campaigns	635	0	2	2	2	2
H2_Testing policy	635	0	1	1	2	3
H3_Contact tracing	635	0	1	1	2	2
H4_Emergency investment in healthcare (1 million USD)	605	0	0	0	0	242000
H5_Investment in vaccines (1 million USD)	631	0	0	0	0	826
**COVID-19 Community Mobility Reports (percent change from baseline)**						
Retail and Recreation	630	-95	-52	-24	-10	18
Grocery and Pharmacy	630	-85	-18	-6	3	59
Parks	630	-91	-30.8	-9	8	92
Transit Stations	630	-89	-59	-31.5	-15	9
Workplaces	630	-84	-45	-14	-3	16
Residential	630	-2	5	9	18	39
**Mobility Trends Reports**						
Walking	635	-1.87	-1.08	-0.76	0.12	2.26
Using public transport	485	-1.33	-1.07	-0.75	0.414	1.97
Driving	635	-1.93	-1.15	-0.77	0.21	2.50

**Fig 1:**
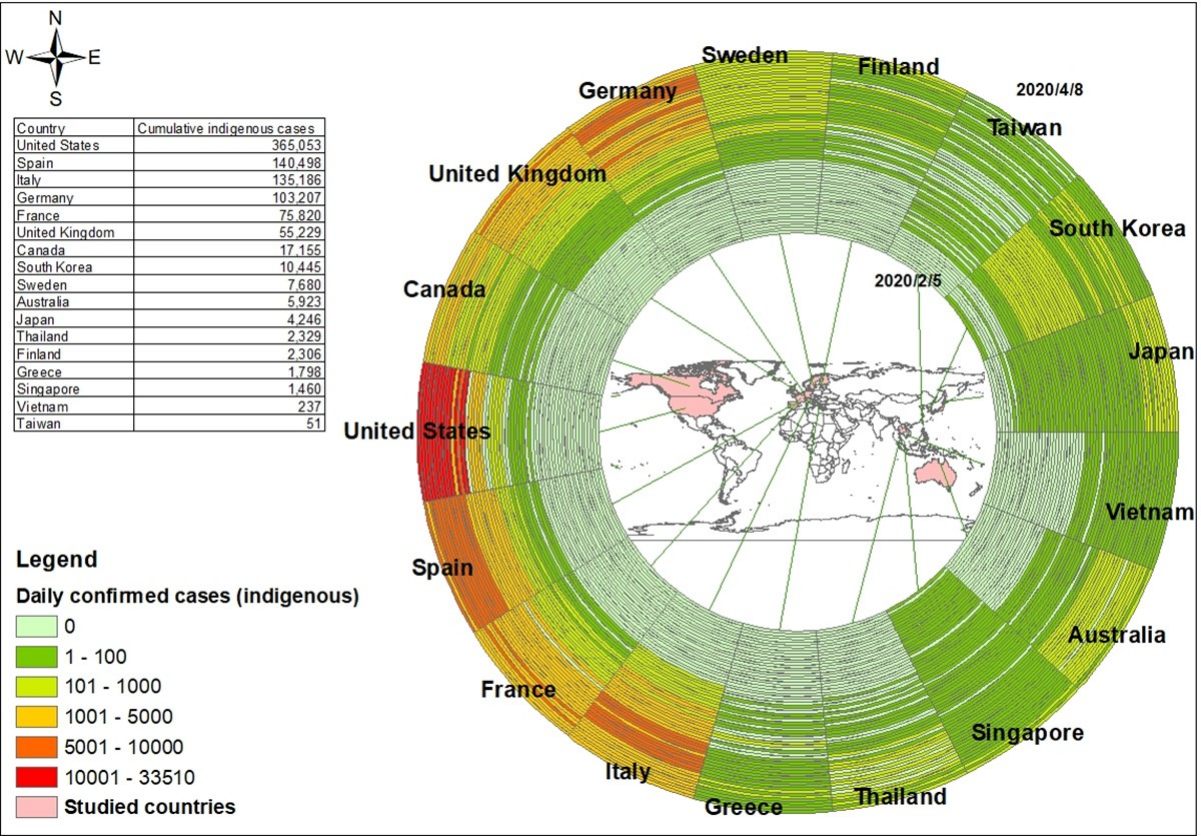
Ring map of daily confirmed local transmission cases of COVID-19, Feb. 5 to Apr. 8, 2020.

**Fig 2:**
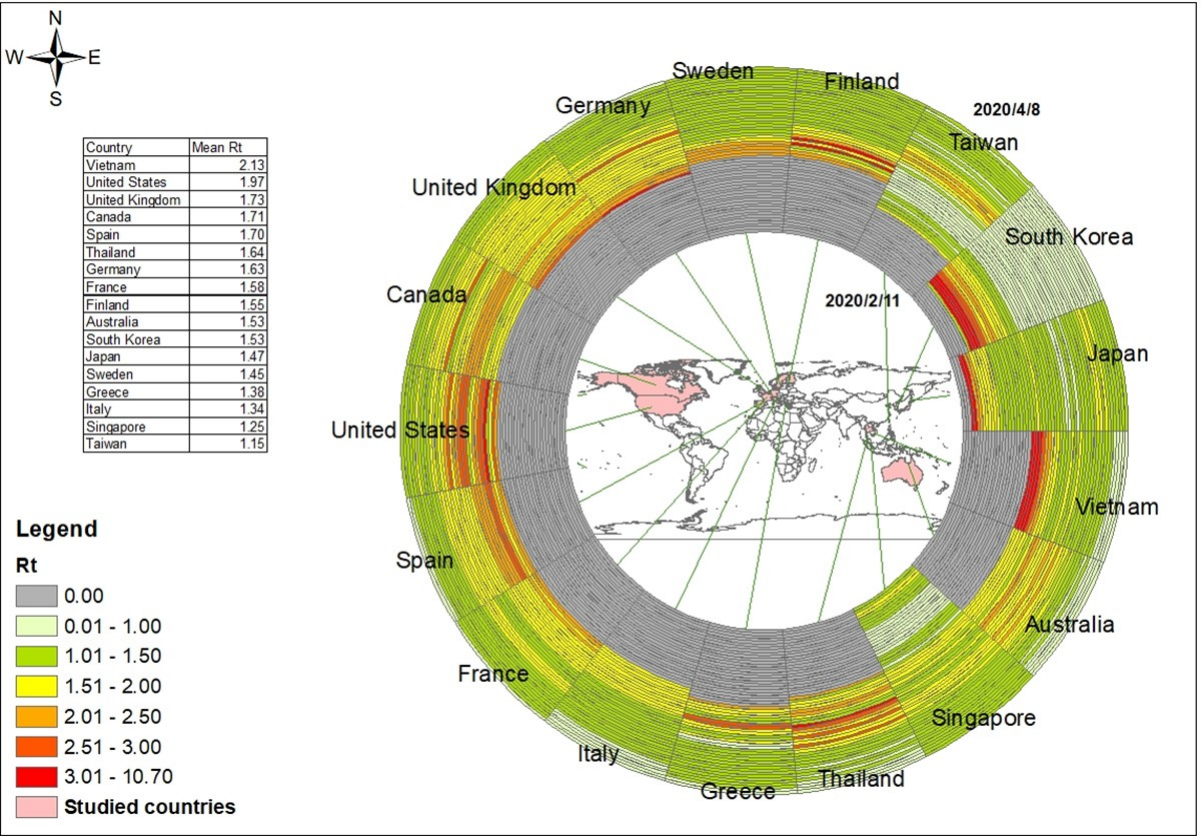
Ring map of daily effective reproduction number (Rt) with 7-day window of COVID-19, Feb. 11 to Apr. 8, 2020.

Our estimates of the time-varying reproduction number Rt are based on daily confirmed cases from local transmission, and the longest estimated period was from February 5 through April 8. Each ring in Fig 1 represents the daily number of local transmission cases in different countries. The temporal order starts from the innermost ring on February 5, 2020 to the outermost ring on April 8, 2020. The number of daily confirmed cases rose gradually, although it decreased in the last period in some places like South Korea (Fig 1). Each ring in Fig 2 represents a 7-day window of the estimated daily effective reproduction number from local transmission cases in different countries. The temporal order starts from the innermost ring on February 11, 2020 to the outermost ring on April 8, 2020. In the first few weeks, there were no local COVID-19 cases in most countries, thus there were no estimated Rt in the rings with gray color. Most countries had high Rt at the beginning and gradually slowed the transmission rate after introducing the NPI policies. For Taiwan, the reproduction number increased to 2.07 on March 20 and decreased to 1.06 on April 8. For the United States, the reproduction number increased to 3.58 over the first week of Marchand decreased to 1.25 in the last week of the study period. During the study period, in Spain the reproduction number increased to a peak of 2.33 before March 14, and then gradually decreased to 0.93 before April 8.

**Fig 3:**
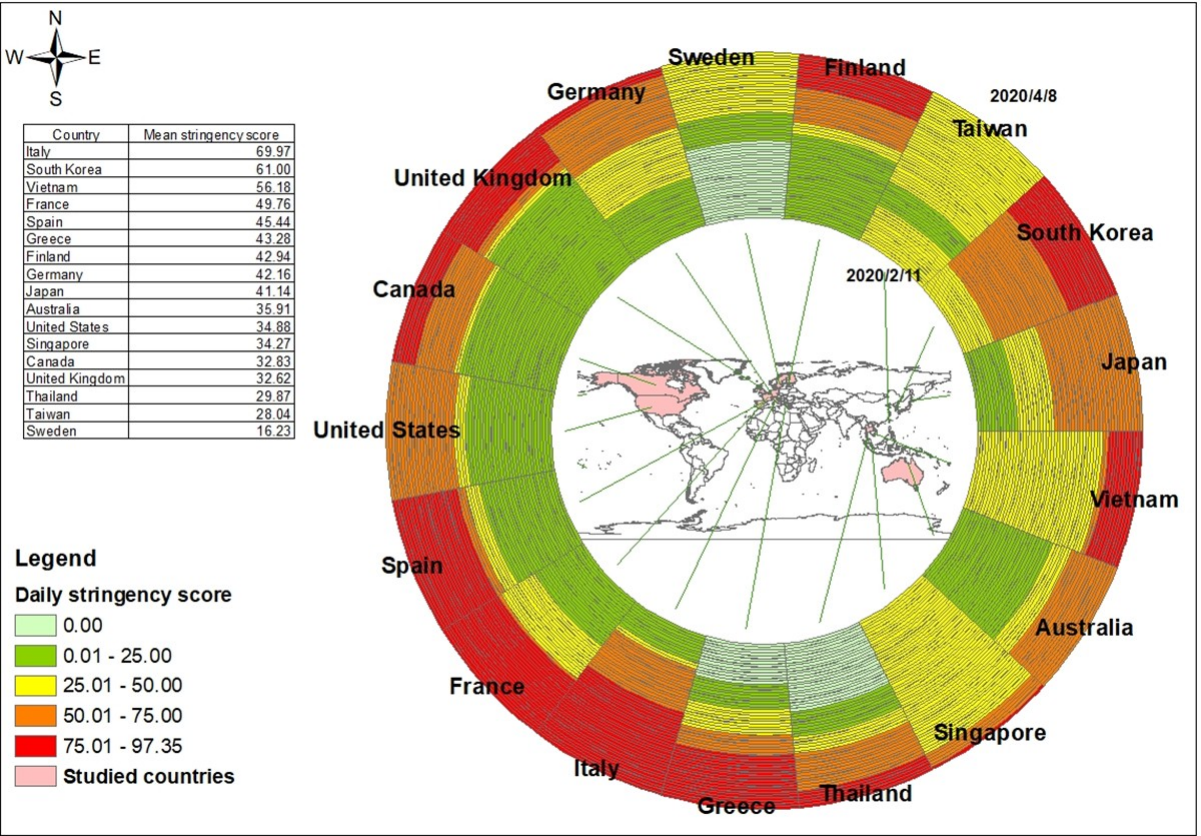
Ring map of daily stringency score from Feb. 11 to Apr. 8, 2020.

**Fig 4:**
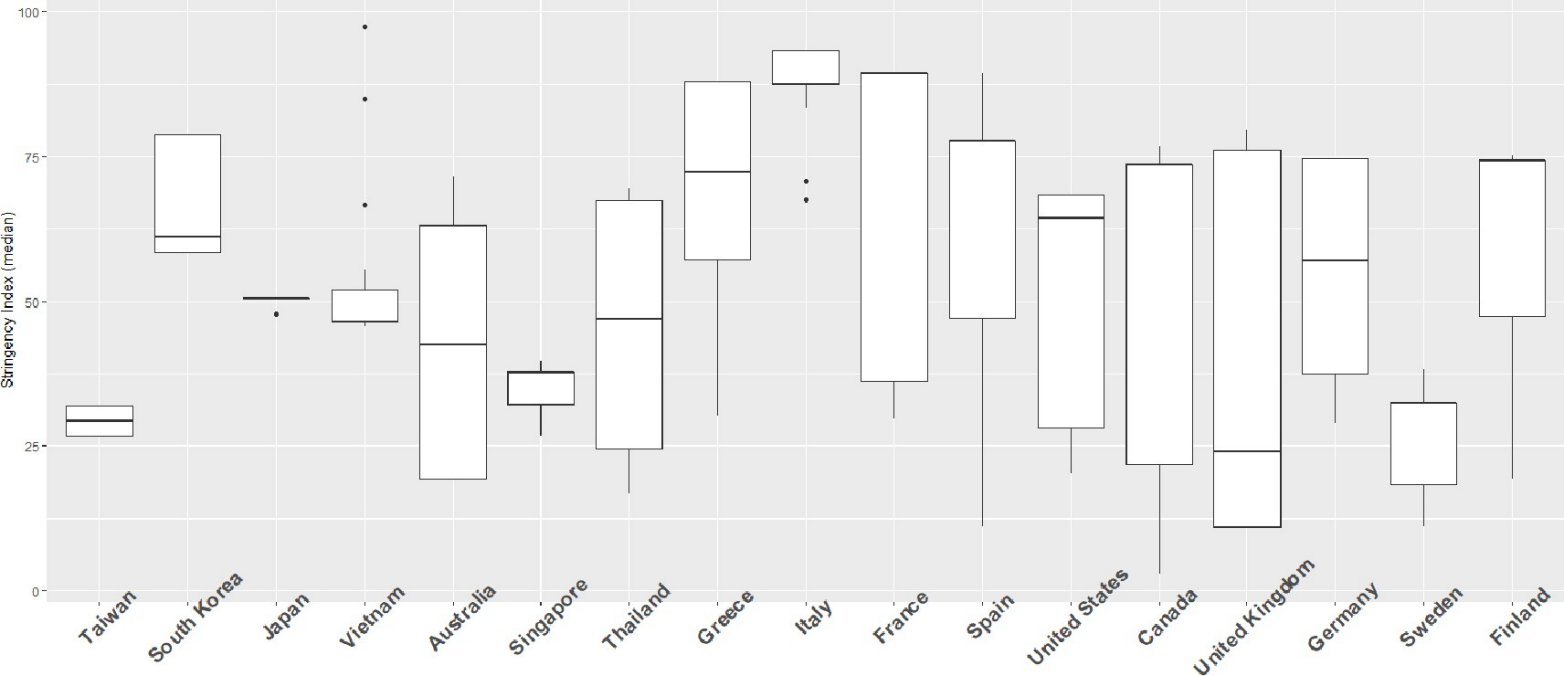
Box plots of stringency scores in 17 countries.

**Fig 5:**
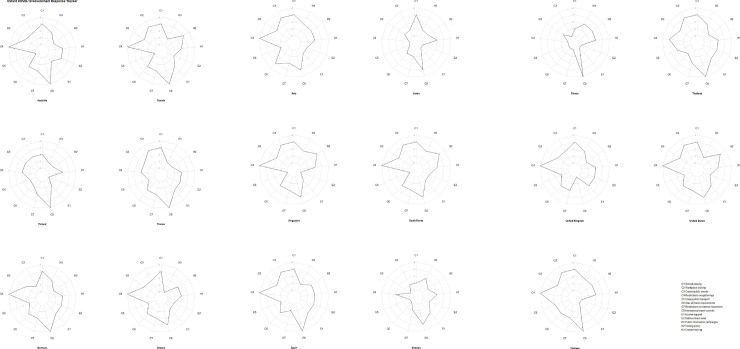
Radar chart of different physical distancing policies with maximum scores in each country.

Each ring in Fig 3 represents the daily stringency score in different countries. The temporal order is from the innermost ring on February 11, 2020 to the outermost ring on April 8, 2020. Among these countries, the strictness of NPIs increased over time during the pandemic, especially in Italy and Spain (Fig 3). In some countries, the stringency was less changed during February 11 to April 8, such as Taiwan and Sweden (Fig 4). The highest two mean policy stringency scores were for Italy (69.97) and South Korea (61.00). Variations in stringency scores were higher in Europe, the US and Australia than in Asia. Regarding the implementation of the stringency policies, these 17 countries had a greater tendency to close schools and universities, limit private gatherings and restrict international travel, and less tendency to shut down public transport in the study period (Table 1, Fig 5). The two groups are classified by the median of the stringency index during the study period. In each region, if the median of the stringency index was below 50, it was classified as lower stringency, otherwise it was classified as higher stringency. The human mobility reduction was greater in countries with strict policies (median stringency score > = 50). And the two groups were significantly different in some community mobility changes including retail and recreation, transit stations, workplaces and residences (Table 2).

**Table 2:** Difference of mobility changes in the countries with high or low stringency index.

	Median of Stringency Index		Estimate	95% CI
Variable	<50	> = 50		
COVID-19 Community Mobility Reports (percent change from baseline)				
Retail and Recreation	-15	-38	-21.73	(-40.83, -2.64)
Grocery and Pharmacy	-5	-8	-9.32	(-22.13, 3.48)
Parks	-10	-7	-8.88	(-39.07, 21.30)
Transit Stations	-22	-46	-17.00	(-32.63, -1.39)
Workplaces	-4	-27	-23.18	(-38.61, -7.76)
Residential	7	12	5.99	(0.07, 11.91)
Mobility Trends Reports				
Walking	-0.44	-0.89	-0.16	(-0.57, 0.25)
Driving	-0.35	-0.94	-0.19	(-0.67, 0.29)

Note: 95% Confidence interval (CI) by random effects model with a random intercept.

**Fig 6:**
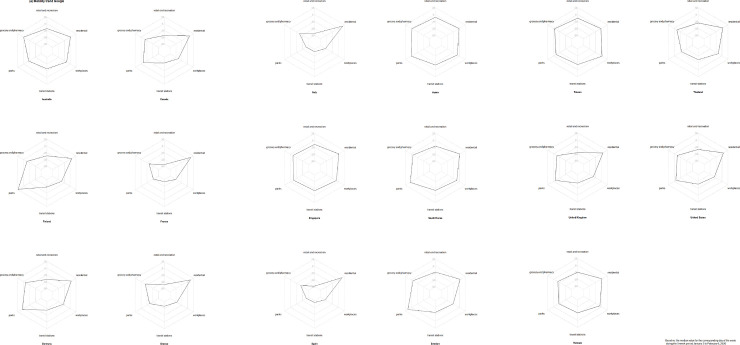
Radar charts of maximum mobility changes during studied period provided by Google.

In the pandemic period, the median of decreases in visits and length of stay was about 24% in retail and recreation, 6% in grocery stores and pharmacies, 9% in parks, 31.5% in transit stations, and 14% in workplaces, while the median increase was 9% in residence compared to the baseline (Table 1, Fig 6). In the countries with stricter NPIs, the behavioral changes were greater. For example, in countries with higher stringency, people visited less often and remained for less time at grocery stores and pharmacies, transit stations, retail and recreation, and workplaces, and stayed at home more (Fig 6).

**Fig 7:**
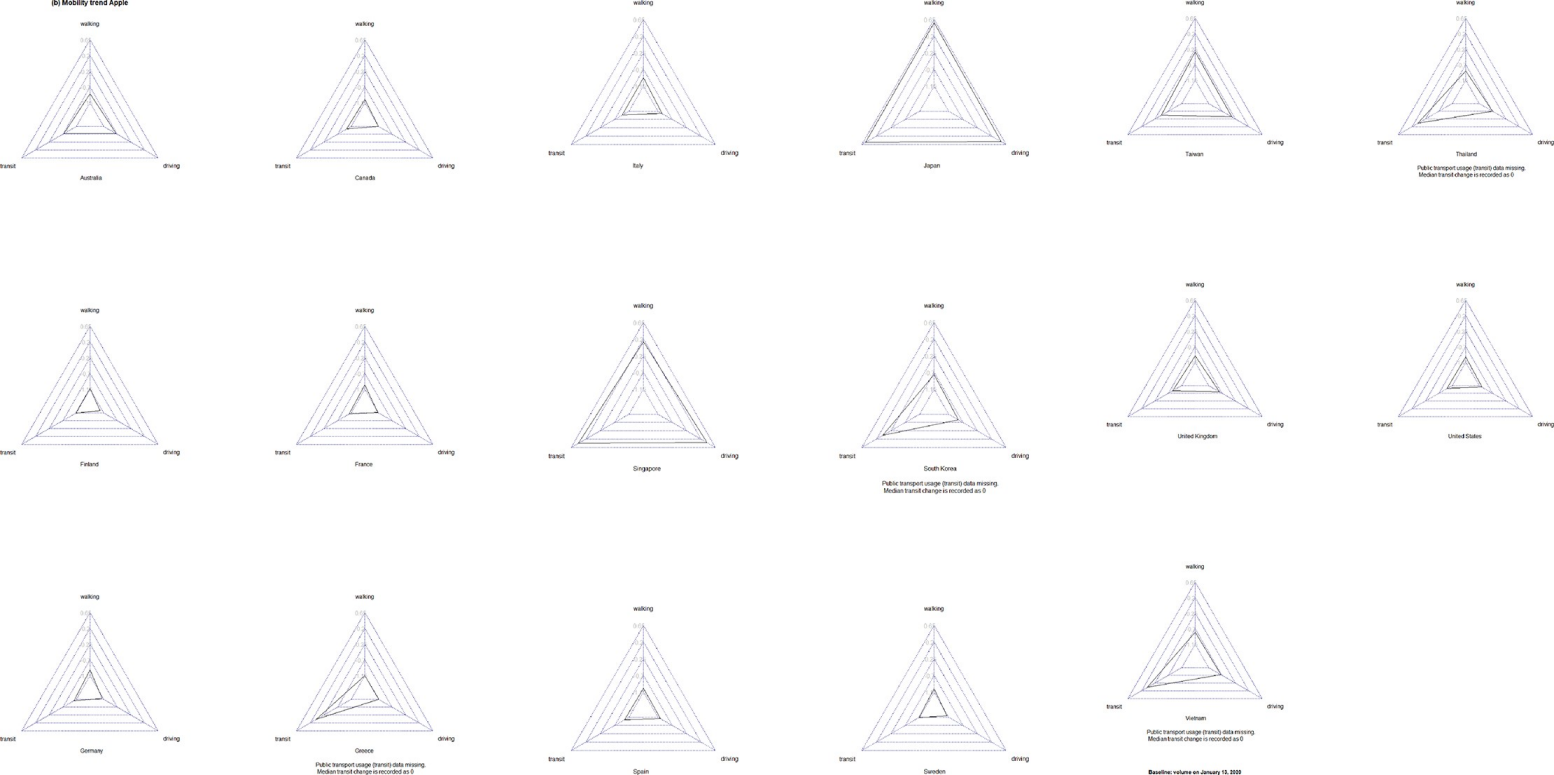
Radar charts of maximum mobility changes during studied period provided by Apple.

The requests for directions by different transportation types in Apple Maps declined in most of the studied countries. Especially in Finland and Sweden, the search times for driving, using public transport and walking to the desired destination were obviously lower than in January 13, 2020 (Fig 7).

While taking the stringency index into account rather than the 11 policies, if the interventions had immediate effect (0-day lagged effect), the percent changes in going to parks, grocery stores and pharmacies, and the changes in requests for directions by walking were significantly associated with the reproduction number. As for the 7-day lagged effect on policies and behavioral change, the results are similar to the 0-day lagged effect, except that there was no significant effect on the percent change for parks. With a 14-day lagged effect on policies and behavioral change, the reproduction number was significantly associated with the stringency index, the percent change to go to workplaces and the changes in requests for directions by walking (Table 3).

**Table 3:** Estimated effects on effective reproduction number.

	0-day lagged effect			7-day lagged effect ^a^		14-day lagged effect ^b^	
Variable	coefficient	95% CI		coefficient	95% CI	coefficient	95% CI
Policy timing	0.009		(-0.002, 0.019)	0.006	(-0.002, 0.015)	0.003	(-0.005, 0.011)
Stringency Index						-0.005	(-0.010, -0.001)*
Grocery and Pharmacy (percent change from baseline)	0.010		(0.005, 0.015)*	0.006	(0.001, 0.010)*		
Parks (percent change from baseline)	-0.006		(-0.009, -0.003)*				
Workplaces (percent change from baseline)				-0.005	(-0.009, 0.000)	-0.005	(-0.010, -0.001)*
Walking	0.370		(0.268, 0.472)*	0.405	(0.319, 0.491)*	0.315	(0.220, 0.410)*
AIC	1520.47			1272.75		1223.63	
Sample size	630			612		591	

Abbreviation: CI = confidence interval.

a: Assumes 7-day lagged effect of policies and behavior change on reproduction number.

b: Assumes 14-day lagged effect of policies and behavior change on reproduction number.

*: p-value< 0.05.

While taking the 11 policies into account, school closures, workplace closures, public event cancellations, restrictions on gatherings, requirements of staying at home, testing policies, the percent change to visits and staying in parks, and the changes in requests for directions by walking were all significantly associated with the reproduction number under an immediate intervention effect. As to the 7-day lagged effect on policies and behavioral change, the results are similar to the 0-day lagged effect, except that the percentage changes in visits and stay in parks were insignificant. Moreover, international arrivals screening, ban on entry from all countries or total border closure, and comprehensive contact tracing were significantly associated with the reproduction number. Looking at the 14-day lagged effect, restrictions on gatherings, contact tracing, the percent change to visits and staying in parks, and changes in requests for directions by walking were significantly associated with the reproduction number (Table 4).

**Table 4:** Estimated effects on effective reproduction number with specific policies.

	0-day lagged effect		7-day lagged effect ^a^		14-day lagged effect ^b^	
Variable	coefficient	95% CI	coefficient	95% CI	coefficient	95% CI
C1 School closing						
recommend closing	0.330	(0.002, 0.658)*	0.265	(-0.059, 0.588)	NA	
require closing (only some levels)	1.242	(0.558, 1.926)*	0.700	(0.099, 1.300)*	NA	
require closing (all levels)	0.871	(0.578, 1.165)*	0.312	(0.068, 0.556)*	NA	
C2 Workplace closing						
recommend work from home	-0.307	(-0.572, -0.043)*	-0.660	(-0.937, -0.382)*	NA	
require closing for some sectors	-0.572	(-0.905, -0.238)*	-0.487	(-0.840, -0.135)*	NA	
require closing for all-but-essential workplaces	-0.656	(-0.986, -0.325)*	-0.290	(-0.656, 0.075)	NA	
C3 Cancel public events						
recommend cancelling	-0.093	(-0.386, 0.200)	0.135	(-0.095, 0.365)	0.003	(-0.192, 0.199)
require cancelling	0.647	(0.342, 0.952)*	0.360	(0.099, 0.621)*	0.159	(-0.060, 0.378)
C4 Restrictions on gatherings						
restrictions on gatherings above 1000 people	-0.088	(-0.467, 0.292)	0.087	(-0.267, 0.441)	-0.299	(-0.590, -0.009)*
restrictions on gatherings between 101–1000 people	-0.421	(-0.712, -0.130)*	-0.320	(-0.594, -0.046)*	-0.377	(-0.634, -0.119)*
restrictions on gatherings between 11–100 people	-0.208	(-0.559, 0.142)	-0.292	(-0.653, 0.070)	-0.566	(-0.931, -0.202)*
restrictions on gatherings of 10 people or less	-0.273	(-0.624, 0.078)	-0.107	(-0.458, 0.243)	-0.325	(-0.684, 0.035)
C6 Requirements of staying at home.						
recommend not leaving house	-1.017	(-1.322, -0.712)*	-0.373	(-0.665, -0.081)*	NA	
require not leaving house with some exceptions	-0.980	(-1.310, -0.650)*	-0.322	(-0.636, -0.008)*	NA	
require not leaving house with minimal exceptions	-1.497	(-2.063, -0.931)*	-0.549	(-1.109, 0.010)	NA	
C7 Restrictions on internal movement						
recommend not traveling	NA		NA		-0.156	(-0.385, 0.074)
internal movement restrictions	NA		NA		-0.030	(-0.310, 0.250)
C8 International travel controls						
screening arrivals	-0.119	(-0.557, 0.319)	-0.258	(-0.644, 0.128)*	-0.197	(-0.505, 0.110)
quarantine arrivals	-0.329	(-0.748, 0.090)	-0.270	(-0.652, 0.113)	-0.113	(-0.393, 0.166)
ban arrivals from some regions	0.141	(-0.207, 0.490)	0.038	(-0.276, 0.352)	0.022	(-0.225, 0.268)
ban on all regions or total border closure	-0.108	(-0.505, 0.288)	-0.260	(-0.655, 0.135)*	-0.253	(-0.602, 0.097)
H1 Public information campaigns ^c^						
coordinated public information campaign	0.304	(-0.199, 0.807)	NA		NA	
H2 Testing policy						
only those who both (a) have symptoms AND (b) meet specific criteria	0.675	(0.206, 1.144)*	0.548	(0.086, 1.011)*	NA	
testing of anyone showing Covid-19 symptoms	0.207	(-0.308, 0.722)	0.490	(-0.071, 1.051)	NA	
open public testing	0.824	(0.249, 1.399)*	0.897	(0.252, 1.543)*	NA	
H3 Contact tracing						
limited contact tracing; not done for all cases	NA ^d^		0.271	(-0.189, 0.731)	0.310	(0.042, 0.577)*
comprehensive contact tracing; done for all identified cases	NA		-0.534	(-0.986, -0.082)*	-0.112	(-0.381, 0.156)
Policy timing	NA		NA		0.003	(-0.004, 0.011)
Workplaces (percent change from baseline)	NA		NA		-0.004	(-0.009, 0.000)
Parks (percent change from baseline)	-0.006	(-0.009, -0.003)*	NA		0.003	(0.000, 0.005)*
Walking	0.255	(0.120, 0.389)*	0.342	(0.227, 0.456)*	0.259	(0.159, 0.358)*
AIC	1463.25		1440.33		1253.34	
Sample size	630		635		591	

abbreviation: CI = confidence interval.

a: Assumes 7-day lagged effect of policies and behavior change on reproduction number.

b: Assumes 14 day lagged effect of policies and behavior change on reproduction number.

c: H1: Records presence of public info campaigns.

coordinated public information campaign (e.g. across traditional and social media).

d: NA for a coefficient indicates that the variable is not selected in the model.

*: p-value< 0.05.

## Discussion

To the best of our knowledge, this is the first paper to evaluate the effects of both the stringency of policies and residents’ compliance on the transmissibility of COVID-19 globally. In our findings, time-varying reproduction numbers surged rapidly at the initial epidemic stage and declined gradually depending on policy stringency. The human mobility reduction was greater in countries with stricter policies. By examining the 0-day, 7-day and 14-day lagged effects of policies and changes in human mobility on Rt, stay-at-home policies, workplace closures, limiting gatherings, international travel controls, contact tracing, less visiting and staying in parks, and reducing walking around were all effective at reducing Rt. In this study, the highest mean time-varying reproduction number was 2.13 in Vietnam, which means an average of 2.13 secondary cases generated by a primary case. Our estimated average time-varying reproduction numbers were 1.34 for Italy and 1.53 for South Korea. Compared to previous studies, the median value of the time-varying reproduction number for Italy’s province of Macerata was 1.792 (1.0–3.5) with the exponential growth (EG) method, estimated with data from February 26, 2020 to April 20, 2020 [18]. The mean estimated reproduction number was 1.5 (1.4–1.6) on February 26, 2020 in South Korea [19].

A portion of COVID-19 cases were imported from other areas, which might lead the reproduction number to be overestimated due to “imported cases” (cases infected abroad who then entered the country, rather than being locally transmitted). Most countries required travelers to fill out a health declaration form, have their temperature measured, undergo self-quarantine and be tested for SARS-CoV-2, the virus causing COVID-19, if necessary. The probability of a potentially infected traveler spreading the disease to healthy local people declines with close confinement. Therefore, this study did not examine imported cases which were unrelated to transmission of local outbreaks.

Moreover, the reproduction number may be underestimated because asymptomatic cases or mild cases may not be detected and reported, especially at the start of an outbreak. Early in the epidemic, Read et al. estimated that the ascertainment rate was only 5.1% in Wuhan [20]. The potential number of infectors was underreported. Furthermore, the reproduction number may change over time based on changing infectivity of the virus, modes of social interaction, effectiveness of interventions and so on [21].

A combination of different non-pharmaceutical interventions has been proved to be useful in reducing the epidemic peak, but the timing, duration and useful combination of different interventions still need to be explored. At the same time, the effectiveness of each intervention is constantly being explored. The case number after intervention policies might be overcounted if the counts include infectees who were still asymptotic and undiagnosed before intervention. Thus, there were lags for the interventions to take effect. We assess the 0-day, 7-day and 14-day lagged effects as a sensitivity analysis. For the 0-day lagged effect scenario, workplace closure, restrictions on gatherings of over a certain number of people (ranging from over 100 to over 1000) and staying at home were effective in reducing the reproduction number. Working at home or work bans were effective when the workplace was a key infection site. Workplace distancing reduced the number of infections in simulation models based on geographical, demographic, and epidemiological data from Singapore [22]. In another study, under other control measures such as bans on public gatherings, prohibition of movement without valid reason, suspension of intra-city public transport and closure of entertainment venues, fewer confirmed cases were reported during the early stage of the outbreak [23]. However, school closures, cancelling public events, testing only those who both have symptoms and meet specific criteria, or open public testing result in increasing, not decreasing, reproduction numbers. The contribution of school closures to transmission control may depend on the features of a disease. There has been limited information about the effectiveness of school closures alone in coronavirus outbreaks such as Severe Acute Respiratory Syndrome (SARS) and Middle East Respiratory Syndrome (MERS). As S1 Table shows, we found an effect from implementing school closure policy alone on reducing the effective reproduction number. However, when we considered other NPI policies together, the effect of school closures disappeared. But for influenza outbreaks, school closures have been shown to be effective. There are several theoretical reasons promoted for the reduced effectiveness of school closures in containing COVID-19 compared to influenza outbreaks [24]. First, relatively few child diagnoses of COVID-19 have been reported worldwide. As of March 8, 2020 the largest proportion of laboratory-confirmed COVID-19 infectees was among the age group of 40–79 in Wuhan, China [25]. Secondly, studies elsewhere have shown that the symptoms are relatively mild in children when compared to adults [26], and the mortality rate is higher in the elderly with underlying diseases [19]. Moreover, when only school closures are implemented, household and community contact tends to increase, such as at day care centers or parks, so the transmission risk does not change much [24]. Alternative measures such as distance learning and closing only confirmed cases’ classes may be considered in the early stage of an outbreak. To sum up, we cannot rule out the possibility that the results of school closure in our study were affected by statistical imprecision such as measurement error, but the reversed effect of school closure in other studies might be related to interactions with other interventions. A policy of testing for SARS-CoV-2 may not work so well if testing is not performed until after onset of symptoms and time is needed to get the test results.

For a 7-day lagged effect scenario, recommendations to work from home, required closure of workplaces for some sectors, restrictions on gatherings of over a certain number of people (ranging from over 100 to over 1000), requirements to stay at home, international travel controls (i.e. screening international airplane arrivals or total border closure), and contact tracing done for all identified cases were all effective in reducing the reproduction number. In a study on travel restrictions in China, the imported cases from Wuhan dropped under a travel ban which prevented people from traveling into and out of Wuhan [17]. The effectiveness of travel restrictions has been supported by studies modeling its effectiveness in preventing the spread of COVID-19, suggesting they are useful in the early stage of an outbreak when cases are limited to a certain area [22]. In the later stages of an epidemic, travel restrictions may be less effective due to the disease already being widespread [17]. The factors causing the reproduction number to rise were similar to those in a 0-day lagged effect scenario. For a 14-day lagged effect scenario, restrictions on gatherings of more than 10 people had the effect of decreasing the reproduction number, but limited contact tracing (not done for all cases) increased the reproduction number. Not tracing for all confirmed cases may be due to the fact that too many cases have been confirmed at the same time and some footprints are too complex to be recalled, resulting in some healthy people coming into contact with the infected person but not being discovered. If we replace the 11 policies with an NPI in our study, the results show that a stricter NPI policy inhibited an increase in the reproduction number, and show that a combination of different NPI policies was useful. People changed their behavior due to interventions and the threat of COVID-19. The same mobility pattern was found in the US from January 1 to April 20, 2020 [27]. These protective behaviors were related to concern over the severity of infection. Studies have shown that people tend to decrease their daily contacts, reduce trips to crowded places, and wear masks when leaving home during COVID-19 outbreaks [28]. People may integrate physical distancing into their lifestyle or postpone some activities until an epidemic wanes. Simulations in other studies have suggested that if people are in a state of panic such as panic-buying in a crowded area, physical distancing is of little use [29]. Using social incentives and support to educate people on the importance of physical distancing would be suitable in this kind of situation to inhibit spread of the virus.

Each policy had its implementation timing, and if the policy was too late or was never implemented, there may be too many nulls in the interaction terms between policy options and the timing of its implementation. Because the timing of each policy was different, we used the earliest policy implementation time as our definition of timing. We considered the interaction term between the stringency index and the timing of the earliest policy implementation. The results in S2 Table showed that the interaction term between the stringency index and the timing of the earliest policy implementation is not significant (p value>0.05). The stringency of the policy and the reduction of human mobility are more important at the earlier stage of pandemic.

In addition, the direct effect from the policy implementation or the human mobility on the Rt in the real world might happen together or have some delays. We examined this issue in two parts, including the effect of the policy on human mobility, and that of human mobility on Rt, and considered possible lag times of 7 days and 14 days. We found an inverse relationship between the stringency index and the change in human mobility, and a positive relationship between lower human mobility and lower Rt. The supplementary results are presented in S1 Appendix.

In our findings, regardless of the length of the lag, leaving home by walking increased the risk of the reproduction number ascending. Adding the times and length staying at parks reveals a risk of increasing the reproduction number at a 14-day lag.

The limitation of our research is that the available time interval for local cases was about 1.5 months. This is because some countries did not clearly define the local and non-local transmission cases in press releases.

In our study, we explored the strength of the implemented interventions, individual behaviors and time-varying reproduction number to analyze the association between intervention, behavioral change and time-varying reproduction number worldwide. We show the effect of each type of intervention on the reproduction number so that authorities can evaluate the effect of different combinations of interventions. Authorities can evaluate the influence on transmission rates, economic activity, vulnerable populations, and substantial costs for society based on empirical experience.

## Conclusion

COVID-19 is an emerging infectious disease which has rapidly become a worldwide crisis. Using early data to estimate the reproduction number can let policy makers know the disease transmission situation, equipping them with information on how to execute interventions early, advise people to pay attention to personal protection, and alter their behavior to prevent the pandemic from expanding further.

## Supporting information

S1 Table Univariate regression of school closure policy versus effective reproduction number. (PDF)

S2 Table Estimated effects on effective reproduction number. (PDF)

S1 Appendix The estimated effects of the stringency of policy on human mobility and of effective reproduction number on human mobility (A: 0-day lag, B: 7-day lag, C: 14-day lag). (A-1) Estimated 0-day lagged stringency index effects on community mobility reports and mobility trends reports; (A-2) Estimated 0-day lagged community mobility and mobility trends effects on effective reproduction number; (B-1) Estimated 7-day lagged stringency index effects on community mobility reports and mobility trends reports; (B-2) Estimated 7-day lagged community mobility and mobility trends effects on effective reproduction number; (C-1) Estimated 14-day lagged stringency index effects on community mobility reports and mobility trends reports; (C-2) Estimated 14-day lagged community mobility and mobility trends effects on effective reproduction number.
(PDF)

## References

[1] Li Q, Guan X, Wu P, et al. Early Transmission Dynamics in Wuhan, China, of Novel Coronavirus-Infected Pneumonia. *New England Journal of Medicine* 2020; 382(13): 1199–207. doi: 10.1056/NEJMoa2001316 31995857 PMC7121484

[2] Jackson C, Vynnycky E, Hawker J, Olowokure B, Mangtani P. School Closures and Influenza: Systematic Review of Epidemiological Studies. *BMJ Open* 2013; 3(2). doi: 10.1136/bmjopen-2012-002149 23447463 PMC3586057

[3] Chung WY, Chiang PS, Luo ST, Lin TY, Tsao KC, Lee MS. A Molecular Approach Applied to Enteroviruses Surveillance in Northern Taiwan, 2008–2012. *PLoS One* 2016; 11(12): e0167532. doi: 10.1371/journal.pone.0167532 27907198 PMC5131993

[4] Flaxman S, Mishra S, Gandy A, et al. Estimating the Effects of Non-Pharmaceutical Interventions on COVID-19 in Europe. *Nature* 2020. doi: 10.1038/s41586-020-2405-7 32512579

[5] Hellewell J, Abbott S, Gimma A, et al. Feasibility of Controlling COVID-19 Outbreaks by Isolation of Cases and Contacts. *Lancet Glob Health* 2020; 8(4): e488–e96. doi: 10.1016/S2214-109X(20)30074-7 32119825 PMC7097845

[6] Adlhoch C, Baka A, Ciotti M, et al. Considerations Relating to Social Distancing Measures in Response to the COVID-19 Epidemic. *European Centre for Disease Prevention and Control* 2020.

[7] Matrajt L, Leung T. Evaluating the Effectiveness of Social Distancing Interventions to Delay or Flatten the Epidemic Curve of Coronavirus Disease. *Emerg Infect Dis* 2020; 26(8). doi: 10.3201/eid2608.201093 32343222 PMC7392458

[8] Long QX, Tang XJ, Shi QL, et al. Clinical and Immunological Assessment of Asymptomatic SARS-CoV-2 Infections. *Nat Med* 2020. doi: 10.1038/s41591-020-0965-6 32555424

[9] Inglesby TV. Public Health Measures and the Reproduction Number of SARS-CoV-2. *JAMA* 2020. doi: 10.1001/jama.2020.7878 32356869

[10] Biggerstaff M, Cauchemez S, Reed C, Gambhir M, Finelli L. Estimates of the Reproduction Number for Seasonal, Pandemic, and Zoonotic Influenza: A Systematic Review of the Literature. *BMC Infect Dis* 2014; 14: 480. doi: 10.1186/1471-2334-14-480 25186370 PMC4169819

[11] Cheng H-Y, Jian S-W, Liu D-P, et al. Contact Tracing Assessment of COVID-19 Transmission Dynamics in Taiwan and Risk at Different Exposure Periods Before and After Symptom Onset. *JAMA Internal Medicine* 2020. doi: 10.1001/jamainternmed.2020.2020 32356867 PMC7195694

[12] Hale T, Sam Webster, Anna Petherick, Toby Phillips, and Beatriz Kira. Oxford COVID-19 Government Response Tracker, Blavatnik School of Government. 2020.

[13] Google LLC. Google COVID-19 Community Mobility Reports. https://www.google.com/covid19/mobility/ Date accessed: May 12, 2020.

[14] Apple. Mobility Trends Reports. https://www.apple.com/covid19/mobility/ Date accessed: May 12, 2020.

[15] Du Z., Xu X., Wu Y., Wang L., Cowling B. J., & Meyers L. Serial Interval of COVID-19 among Publicly Reported Confirmed Cases. *Emerging Infectious Diseases* 2020; 26(6), 1341–1343. doi: 10.3201/eid2606.200357 32191173 PMC7258488

[16] Cori A, Ferguson NM, Fraser C, Cauchemez S. A New Framework and Software to Estimate Time-Varying Reproduction Numbers during Epidemics. *American Journal of Epidemiology* 2013; 178(9): 1505–12. doi: 10.1093/aje/kwt133 24043437 PMC3816335

[17] Kraemer MUG, Yang CH, Gutierrez B, et al. The Effect of Human Mobility and Control Measures on the COVID-19 Epidemic in China. *Science* 2020; 368(6490): 493–7. doi: 10.1126/science.abb4218 32213647 PMC7146642

[18] Chintalapudi N, Battineni G, Sagaro GG, Amenta F. COVID-19 Outbreak Reproduction Number Estimations and Forecasting in Marche, Italy. *International Journal of Infectious Diseases* 2020; 96: 327–33. doi: 10.1016/j.ijid.2020.05.029 32437930 PMC7211603

[19] Shim E, Tariq A, Choi W, Lee Y, Chowell G. Transmission Potential and Severity of COVID-19 in South Korea. *International Journal of Infectious Diseases* 2020; 93: 339–44. doi: 10.1016/j.ijid.2020.03.031 32198088 PMC7118661

[20] Read JM, Bridgen JR, Cummings DA, Ho A, Jewell CP. Novel Coronavirus 2019-nCoV: Early Estimation of Epidemiological Parameters and Epidemic Predictions. *MedRxiv* 2020. 10.1098/rstb.2020.0265 PMC8165596 34053269

[21] Thompson RN, Stockwin JE, van Gaalen RD, et al. Improved Inference of Time-Varying Reproduction Numbers during Infectious Disease Outbreaks. *Epidemics* 2019; 29: 100356. doi: 10.1016/j.epidem.2019.100356 31624039 PMC7105007

[22] Park M, Cook AR, Lim JT, Sun Y, Dickens BL. A Systematic Review of COVID-19 Epidemiology Based on Current Evidence. *Journal of Clinical Medicine* 2020; 9(4). doi: 10.3390/jcm9040967 32244365 PMC7231098

[23] Tian H, Liu Y, Li Y, et al. An Investigation of Transmission Control Measures during the First 50 Days of the COVID-19 Epidemic in China. 2020; 368(6491): 638–42. 10.1126/science.abb6105 PMC7164389 32234804

[24] Viner RM, Russell SJ, Croker H, et al. School Closure and Management Practices during Coronavirus Outbreaks including COVID-19: A Rapid Systematic Review. *The Lancet Child & Adolescent Health* 2020; 4(5): 397–404. doi: 10.1016/S2352-4642(20)30095-X 32272089 PMC7270629

[25] Pan A, Liu L, Wang C, et al. Association of Public Health Interventions with the Epidemiology of the COVID-19 Outbreak in Wuhan, China. *JAMA* 2020; 323(19): 1–9. doi: 10.1001/jama.2020.6130 32275295 PMC7149375

[26] Balasubramanian S, Rao NM, Goenka A, Roderick M, Ramanan AV. Coronavirus Disease 2019 (COVID-19) in Children—What We Know So Far and What We Do Not. *Indian Pediatrics* 2020; 57(5): 435–42. doi: 10.1007/s13312-020-1819-5 32273490 PMC7240240

[27] Badr HS, Du H, Marshall M, Dong E, Squire MM, Gardner LM. Association between Mobility Patterns and COVID-19 Transmission in the USA: A Mathematical Modelling Study. *The Lancet Infectious Diseases* 2020. doi: 10.1016/S1473-3099(20)30553-3 32621869 PMC7329287

[28] Zhang J, Litvinova M, Liang Y, et al. Changes in Contact Patterns Shape the Dynamics of the COVID-19 Outbreak in China. *Science* 2020; 368(6498): 1481–6. doi: 10.1126/science.abb8001 32350060 PMC7199529

[29] Bouchnita A, Jebrane A. A Hybrid Multi-Scale Model of COVID-19 Transmission Dynamics to Assess the Potential of Non-Pharmaceutical Interventions. *Chaos*, *Solitons & Fractals* 2020; 138: 109941. doi: 10.1016/j.chaos.2020.109941 32834575 PMC7269965

