# Supplementary material for: Impact of physical distancing policy on reducing transmission of SARS-CoV-2 globally: Perspective from government’s response and residents’ compliance
Source: PLoS One. 2021 Aug 10;16(8):e0255873. doi: 10.1371/journal.pone.0255873 (PMC8354459; doi:10.1371/journal.pone.0255873)
Supplement: S1 Table — (PDF) [file pone.0255873.s001.pdf]

S1 Table. Univariate regression of school closure policy versus effective reproduction number

Variable	coefficient	95% CI
C1 School closing		
recommend closing	-0.06	(-0.41 , 0.29)
require closing (only some levels)	-0.70	(-1.25 , -0.15) *
require closing (all levels)	-0.31	(-0.52 , -0.10) *

*: p-value< 0.05
