# Supplementary material for: Impact of physical distancing policy on reducing transmission of SARS-CoV-2 globally: Perspective from government’s response and residents’ compliance
Source: PLoS One. 2021 Aug 10;16(8):e0255873. doi: 10.1371/journal.pone.0255873 (PMC8354459; doi:10.1371/journal.pone.0255873)
Supplement: S2 Table — (PDF) [file pone.0255873.s002.pdf]

S2 Table. Estimated effects on effective reproduction number

	0-day lagged effect			7-day lagged effect [a]			14-day lagged effect [b]		
Variable	coefficient	95% CI		coefficient	95% CI		coefficient	95% CI	
Policy timing	0.014	(-0.001 , 0.029)		0.009	(-0.001 , 0.02)		0.006	(-0.004 , 0.015)	
Stringency Index	-0.0104	(-0.017 , -0.004)	*	-0.003	(-0.008 , 0.003)		-0.004	(-0.009 , 0.001	
Grocery and Pharmacy (percent change from baseline)	0.00851	(0.003 , 0.014)	*	0.006	(0.001 , 0.011)	*	0.001	(-0.004 , 0.007)	
Parks (percent change from baseline)	-0.0057	(-0.009 , -0.002)	*	-0.001	(-0.004 , 0.001)		0.002	(-0.001 , 0.005)	
Workplaces (percent change from baseline)	-0.0055	(-0.012 , 0.001)		-0.006	(-0.011 , 0)	*	-0.006	(-0.012 , -0.001)	*
Walking	0.25327	(0.119 , 0.387)	*	0.367	(0.264 , 0.47)	*	0.302	(0.206 , 0.399)	*
Policy timing*Stringency Index	-0.0002	(-0.0004 , 0.00004)		-0.0001	(-0.0003 , 0.0001)		-0.0001	(-0.0003 , 0.0001)	
AIC	1546.46			1312.23			1263.9		
Sample size	630			612			591		

Abbreviation: CI= confidence interval

a: Assumes 7-day lagged effect of policies and behavior change on reproduction number

b: Assumes 14-day lagged effect of policies and behavior change on reproduction number

*: p-value< 0.05
