# Supplementary material for: Impact of physical distancing policy on reducing transmission of SARS-CoV-2 globally: Perspective from government’s response and residents’ compliance
Source: PLoS One. 2021 Aug 10;16(8):e0255873. doi: 10.1371/journal.pone.0255873 (PMC8354459; doi:10.1371/journal.pone.0255873)
Supplement: S1 Appendix — The estimated effects of the stringency of policy on human mobility and of effective reproduction number on human mobility (A: 0-day lag, B: 7-day lag, C: 14-day lag). (A-1) Estimated 0-day lagged stringency index effects on community mobility reports and mobility trends reports; (A-2) Estimated 0-day lagged community mobility and mobility trends effects on effective reproduction number; (B-1) Estimated 7-day lagged stringency index effects on community mobility reports and mobility trends reports; (B-2) Estimated 7-day lagged community mobility and mobility trends effects on effective reproduction number; (C-1) Estimated 14-day lagged stringency index effects on community mobility reports and mobility trends reports; (C-2) Estimated 14-day lagged community mobility and mobility trends effects on effective reproduction number. (PDF) [file pone.0255873.s003.pdf]

S1 Appendix. The estimated effects of the stringency of policy on human mobility and of effective reproduction number on human mobility (A: 0-day lag, B: 7-day lag, C: 14-day lag)

(A-1) Estimated 0-day lagged stringency index effects on community mobility reports and mobility trends reports

	Retail and Recreation	
	coefficient	95% CI
stringency index	-0.957	(-0.999 , -0.915)
	Grocery and Pharmacy	
	coefficient	95% CI
stringency index	-0.552	(-0.602 , -0.501)
	Parks	
	coefficient	95% CI
stringency index	-0.550	(-0.635 , -0.464)
	Transit Stations	
	coefficient	95% CI
stringency index	-0.961	(-0.997 , -0.925)
	Workplaces	
	coefficient	95% CI
stringency index	-0.821	(-0.862 , -0.780)
	Residential	
	coefficient	95% CI
stringency index	0.331	(0.315 , 0.348)
	Walking	
	coefficient	95% CI
stringency index	-0.028	(-0.030 , -0.026)
	Driving	
	coefficient	95% CI
stringency index	-0.030	(-0.031 , -0.028)

Abbreviation: CI= confidence interval

Univariate regression.

(A-2)Estimated 0-day lagged community mobility and mobility trends effects on effective reproduction number

	0-day lagged effect	
Variable	coefficient	95% CI
**COVID-19 Community Mobility Reports (percent change from baseline)**		
Retail and Recreation	0.012	(0.009 , 0.014)
Grocery and Pharmacy	0.012	(0.009 , 0.016)
Parks	0.003	(0.0002 , 0.005)
Transit Stations	0.012	(0.009 , 0.015)
Workplaces	0.011	(0.008 , 0.014)
Residential	-0.029	(-0.037 , -0.021)
**Mobility Trends Reports**		
Walking	0.402	(0.316 , 0.488)
Driving	0.371	(0.287 , 0.455)

Abbreviation: CI= confidence interval

Univariate regression.

(B-1) Estimated 7-day lagged stringency index effects on community mobility reports and mobility trends reports

	Retail and Recreation		
		coefficient	95% CI
stringency index		-0.919	(-0.958 , -0.881)
	Grocery and Pharmacy		
		coefficient	95% CI
stringency index		-0.462	(-0.494 , -0.430)
	Parks		
		coefficient	95% CI
stringency index		-0.583	(-0.647 , -0.520)
	Transit Stations		
		coefficient	95% CI
stringency index		-0.942	(-0.978 , -0.906)
	Workplaces		
		coefficient	95% CI
stringency index		-0.735	(-0.768 , -0.701)
	Residential		
		coefficient	95% CI
stringency index		0.310	(0.296 , 0.323)
	Walking		
		coefficient	95% CI
stringency index		-0.030	(-0.032 , -0.029)
	Driving		
		coefficient	95% CI
stringency index		-0.032	(-0.034 , -0.030)

Abbreviation: CI= confidence interval

Univariate regression.

(B-2) Estimated 7-day lagged community mobility and mobility trends effects on effective reproduction number

	7-day lagged effect	
Variable	coefficient	95% CI
**COVID-19 Community Mobility Reports (percent change from baseline)**		
Retail and Recreation	0.012	(0.010 , 0.014)
Grocery and Pharmacy	0.012	(0.009 , 0.016)
Parks	0.006	(0.003 , 0.008)
Transit Stations	0.013	(0.010 , 0.015)
Workplaces	0.011	(0.009 , 0.014)
Residential	-0.031	(-0.038 , -0.025)
**Mobility Trends Reports**		
Walking	0.433	(0.365 , 0.501)
Driving	0.412	(0.345 , 0.480)

Abbreviation: CI= confidence interval

Univariate regression.

(C-1) Estimated 14-day lagged stringency index effects on community mobility reports and mobility trends reports

Retail and Recreation		
	coefficient	95% CI
stringency index	-0.910	(-0.970 , -0.851)
Grocery and Pharmacy		
	coefficient	95% CI
stringency index	-0.448	(-0.491 , -0.405)
Parks		
	coefficient	95% CI
stringency index	-0.558	(-0.638 , -0.479)
Transit Stations		
	coefficient	95% CI
stringency index	-0.922	(-0.981 , -0.864)
Workplaces		
	coefficient	95% CI
stringency index	-0.738	(-0.787 , -0.688)
Residential		
	coefficient	95% CI
stringency index	0.310	(0.290 , 0.330)
Walking		
	coefficient	95% CI
stringency index	-0.029	(-0.032 , -0.027)

Driving		
	coefficient	95% CI
stringency index	-0.031	(-0.033 , -0.028)

Abbreviation: CI= confidence interval

Univariate regression.

(C-2) Estimated 14-day lagged community mobility and mobility trends effects on effective reproduction number

	14-day lagged effect	
Variable	coefficient	95% CI
**COVID-19 Community Mobility Reports (percent change from baseline)**		
Retail and Recreation	0.011	(0.009 , 0.014)
Grocery and Pharmacy	0.009	(0.005 , 0.013)
Parks	0.007	(0.004 , 0.009)
Transit Stations	0.012	(0.010 , 0.015)
Workplaces	0.010	(0.007 , 0.013)
Residential	-0.032	(-0.039 , -0.024)
**Mobility Trends Reports**		
Walking	0.377	(0.307 , 0.446)
Driving	0.362	(0.291 , 0.432)

Abbreviation: CI= confidence interval

Univariate regression.
